# Predicting the holistic force-displacement relation of the periodontal ligament: in-vitro experiments and finite element analysis

**DOI:** 10.1186/1475-925X-13-107

**Published:** 2014-07-30

**Authors:** Chih-Han Chang, Yao-Ning Lei, Yi-Hung Ho, Yu-Hsing Sung, Ting-Sheng Lin

**Affiliations:** 1 Institute of Biomedical Engineering, National Cheng Kung University, Tainan 701, Taiwan; 2 Department of Dentistry, E-Da Hospital, Kaohsiung 824, Taiwan; 3 Department of Biomedical Engineering, I-Shou University, Kaohsiung 824, Taiwan

**Keywords:** Micro-computed tomography, Finite element analysis, Periodontal ligament

## Abstract

**Background:**

The biomechanical property of the periodontal ligament (PDL) is important in orthodontics and prosthodontics. The objective of this study was to evaluate the feasibility of measuring the biomechanical behavior of the periodontal ligament using micro-computed tomography (micro-CT).

**Methods:**

A custom-made apparatus measured the force and displacement of a porcine PDL specimen within the micro-CT environment. Synchronized computed tomography (CT) images were used to obtain the deformation and displacement of the entire specimen and to reconstruct the three-dimensional mesh model. To match the experimental results, finite element analysis was then applied to simulate the biomechanical response of the PDL. The mechanical model of the PDL was assumed as the hyperelastic material in this study.

**Results:**

The volume variations of the tooth and the alveolar bone were less than 1%, which implies that tooth displacement was caused mostly by displacement of the PDL. Only translational displacement was observed with each load step because the transformation matrix acquired from the CT image registration was identical. The force-displacement curve revealed the nonlinear behavior of the PDL. There was a high correlation between the experimental displacement results and the simulation displacement results. The numerical results (based on the assumption that the PDL is the hyperelastic material) showed good agreement with the experimental results.

**Conclusions:**

Nondestructive measurements by micro-CT obtained the biomechanical behavior of the PDL. Using the hyperelastic characteristic as the constitutive model can properly predict the force-displacement relation of the PDL after loading. This study provided a feasible approach for measuring the biomechanical behavior of the PDL for further dental application.

## Background

The periodontal ligament (PDL) has a determinative role in dental biomechanics. The PDL connects the tooth and alveolar bone, absorbs occlusal impact, and forms and resorbs the alveolar bone for tooth movement. Hence, the biomechanical property of the PDL is important in orthodontics and prosthodontics. Orthodontic treatment is a reiterative and time-consuming process during which a tooth gradually moves to its prospective position. The physiological mechanism of tooth movement is primarily because of the response of the PDL while an orthodontic force is applied [1].

The PDL is clearly important, but its biomechanical behavior remains unclear. Toms et al. [2] performed a shear test by using a transverse thin specimen of the premolar tooth. They revealed the anisotropic behavior of the PDL. Dorow et al. [3] designed an apparatus to test the response of the porcine anterior tooth under different strain rates. Their results showed that the nonlinear hysteresis behavior varied with the strain rate. Natali et al. [4] designed a laser-optical system to measure the minipig premolar tooth. They obtained the nonlinear force-displacement relation of the PDL. In 2009, Tohill et al. [5] acquired the relaxation characteristic of the premolar porcine tooth. However, these studies only demonstrated the simplex mechanical feature of the PDL.

To date, the PDL cannot be harvested by a nondestructive approach for evaluating the stress–strain relation. Analytical models have consequently become a popular approach for estimating the biomechanical behavior of the PDL. There are three types of governing equation for these models: (1) linear elastic model, (2) viscoelastic model, and (3) hyperelastic model. The linear elastic behavior of the PDL has been widely introduced in previous literature reports, but the Young’s modulus values range from 0.07 MPa to 1750 MPa [6]. However, some studies indicate that the elastic property of the PDL is nonlinear [7-9]. Previous analytical models have assumed viscoelastic properties for the PDL such as creep, stress relaxation, and hysteresis [10-13]. However, most PDL researchers prefer the linear elastic behavior because of its simplicity. Because the force-displacement relation of the PDL is insufficient, an energy-based equation was developed and named the “hyperelastic model”. To predict tooth mobility or movement, many researchers have tried to establish a hyperelastic model of the PDL [4,13-15]. Choosing hyperelastic models and parameters is arduous and may directly influence the simulation results. The dilemma of predicting PDL behavior has limited the advancement of dental biomechanics.

Micro-computed tomography has recently been widely applied for dental research in the microscale such as dental morphology [16], dental biology [17], dental biomechanics [18,19], and dental material science [20]. This method can detect the precise morphology and can inspect the details of the microstructure or microvariations (e.g., movement or deformation) in biological tissues. Therefore, we aimed to measure the biomechanical behavior of the PDL using micro-CT.

## Methods

**Figure 1:**
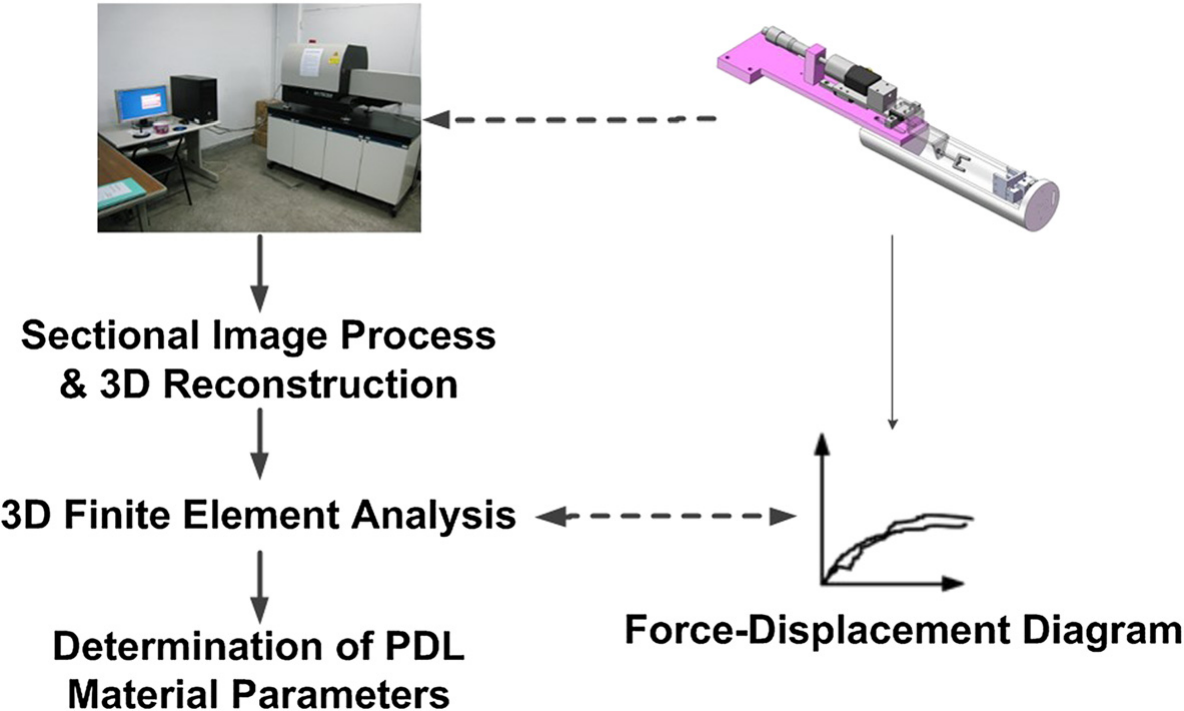
The research protocol of the study.

Figure 1 shows our research protocol. A porcine premolar tooth with the mandible was harvested for biomechanical testing. A device was designed to synchronously measure the reaction force under controlled displacement in the micro-CT environment. The sectional micro-CT images were simultaneously digitized for three-dimensional (3D) FE model reconstruction. The force-displacement diagrams of the FE and the experimental results were compared to determine the material parameters of the PDL.

### Custom device for installation into the micro-CT scanner

**Figure 2:**
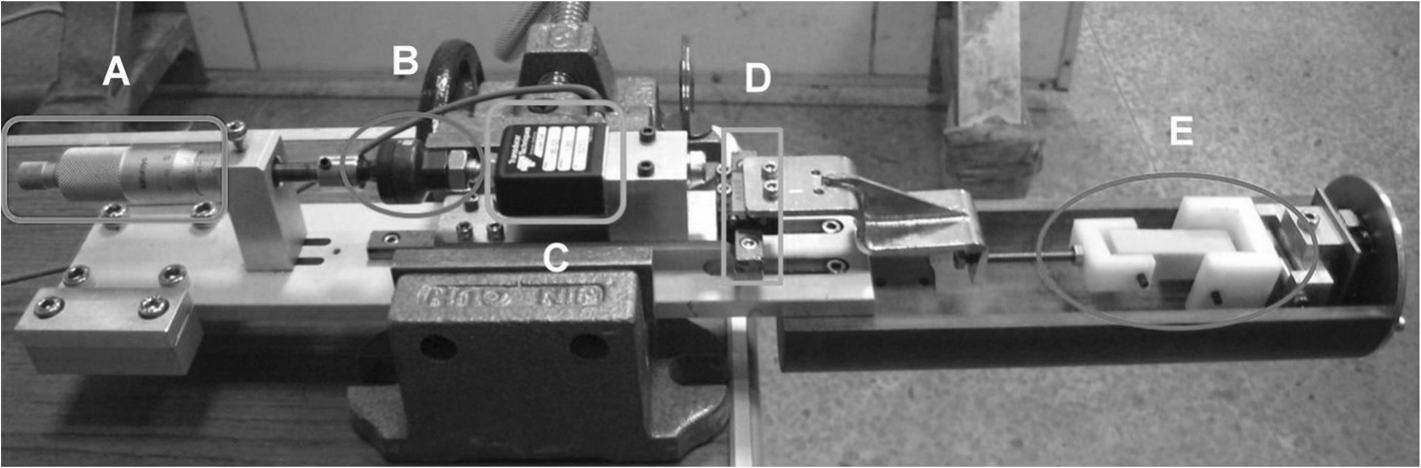
**The custom-made apparatus. (A)** micrometer; **(B)** universal joint; **(C)** load cell; **(D)** linear motion rolling guide; and **(E)** sample jig.

For simultaneously obtaining the reaction force and the displacement of the entire premolar tooth, a device was developed for the micro-CT environment. The device was able to control the displacement and to measure the reaction force. As shown in Figure 2, the main components of the device were (1) a micrometer (Model No. 103–137, Mitutoyo Corporation., Japan) with a sensitivity of 0.01 mm to control the displacement of the premolar tooth in our experiment; (2) a universal joint (JA20-8-125 floating joint; SMC Corporation, IN, USA) to ensure that the direction of the reaction force would remain axially loaded; (3) a load cell (MDB-50; Transducer Techniques, CA, USA) to record the magnitude of the axial force; (4) a linear motion rolling guide (IKO LWL9 B; Nippon Thompson Co., Ltd., Japan) in both directions: first, to maintain the same direction between the load cell and the micrometer and, second, to drive the sample jig to apply tension or a compression force to the specimen; and (5) the sample jig to fix the specimen and apply force. The entire device was designed based on the folder size of micro-CT. It could record the force applied to the tooth.

### Experiment

**Figure 3:**
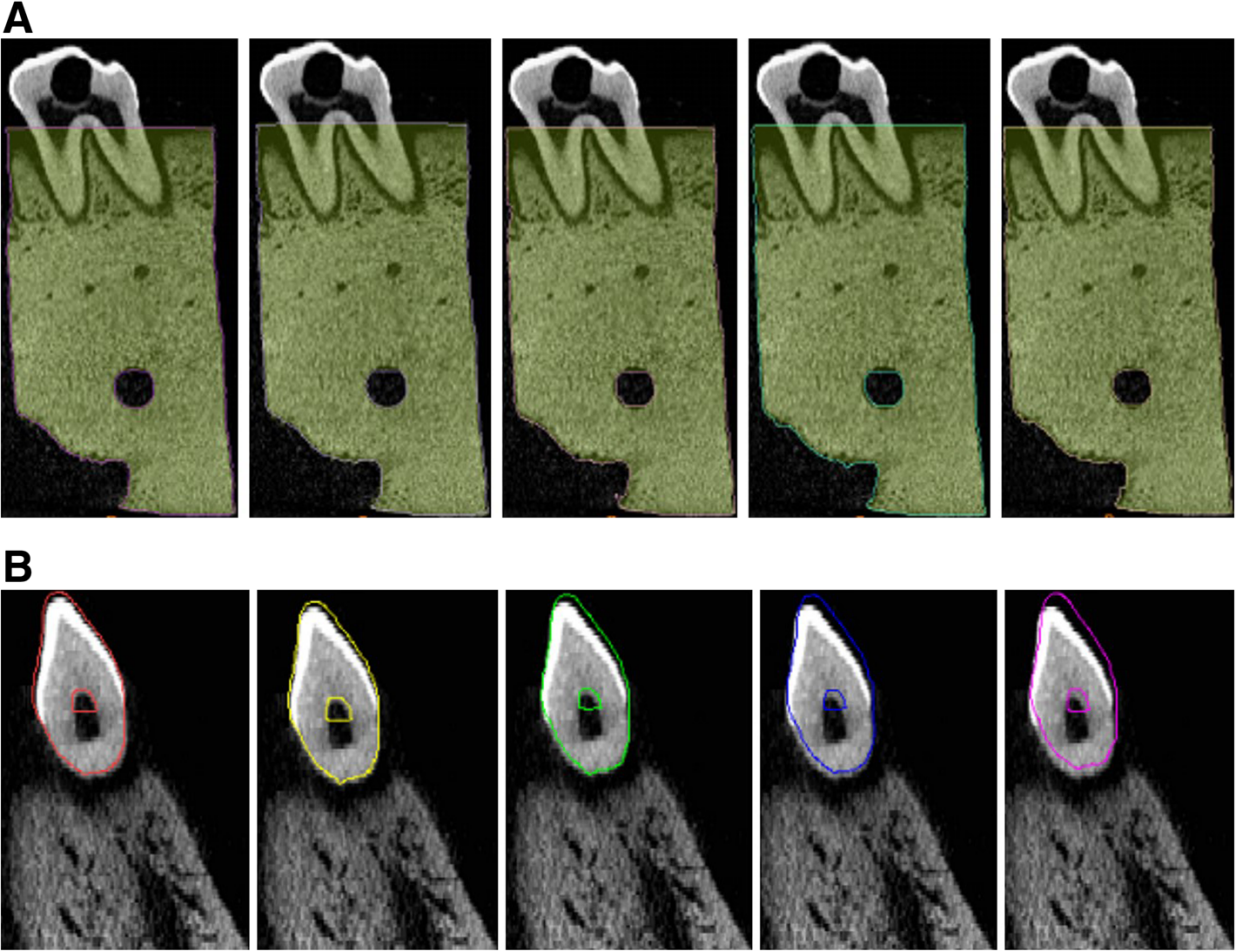
**The STL registration results of the alveolar bone and tooth (applied displacement from left to right: 0.17 mm, 0.34 mm, 0.60 mm, 0.84 mm and 1.28 mm). (A)** Colored contour represented that the postloaded alveolar bone was superimposed to the preloaded bone. **(B)** Colored contour represented that the postloaded tooth was superimposed to the preloaded tooth.

A porcine premolar tooth was harvested, but the surrounding gingiva and mucosa were eliminated. The complete specimen included the premolar tooth, PDL, and alveolar bone. The crown and alveolar bone were both perforated with a 3-mm hole to fix them into the sample jig. The premolar tooth was then loaded by micrometer with constant displacements of 0.17 mm, 0.34 mm, 0.60 mm, 0.84 mm, and 1.28 mm while the reaction force was recorded in the end of the micro-CT scan procedure. Sectional images of the entire specimen were scanned by micro-CT (SkyScan 1076; Bruker-MicroCT, Belgium) for further image processing and for the reconstruction of the finite element model. The parameters of the micro-CT were 70 kV, 100 μA, 35-μm resolution, and 316-ms exposure time.The real displacement of the premolar tooth was calculated from the preloaded and postloaded stereolithography (STL) model that was reconstructed from micro-CT images by medical image software (Mimics v15.01; Materialise, Belgium). The preloaded alveolar bone and tooth models were respectively reconstructed as the references. The perforated holes in the alveolar bone and tooth were selected as the registration points. By superimposing the postloaded alveolar bone model, the displacement of the tooth could be obtained (Figure 3). During image processing, the alveolar bone was assumed to be a rigid body (i.e., the alveolar bone had no deformation or displacement). A built-in function (i.e., STL registration) was adopted to compare the volumes of the alveolar bone and tooth to ensure that variation was mostly caused by the displacement of the tooth, rather than by bone deformation. In addition, a 4 × 4 transformation matrix could be obtained as the real displacement of the premolar tooth.

### Finite element analysis

**Figure 4:**
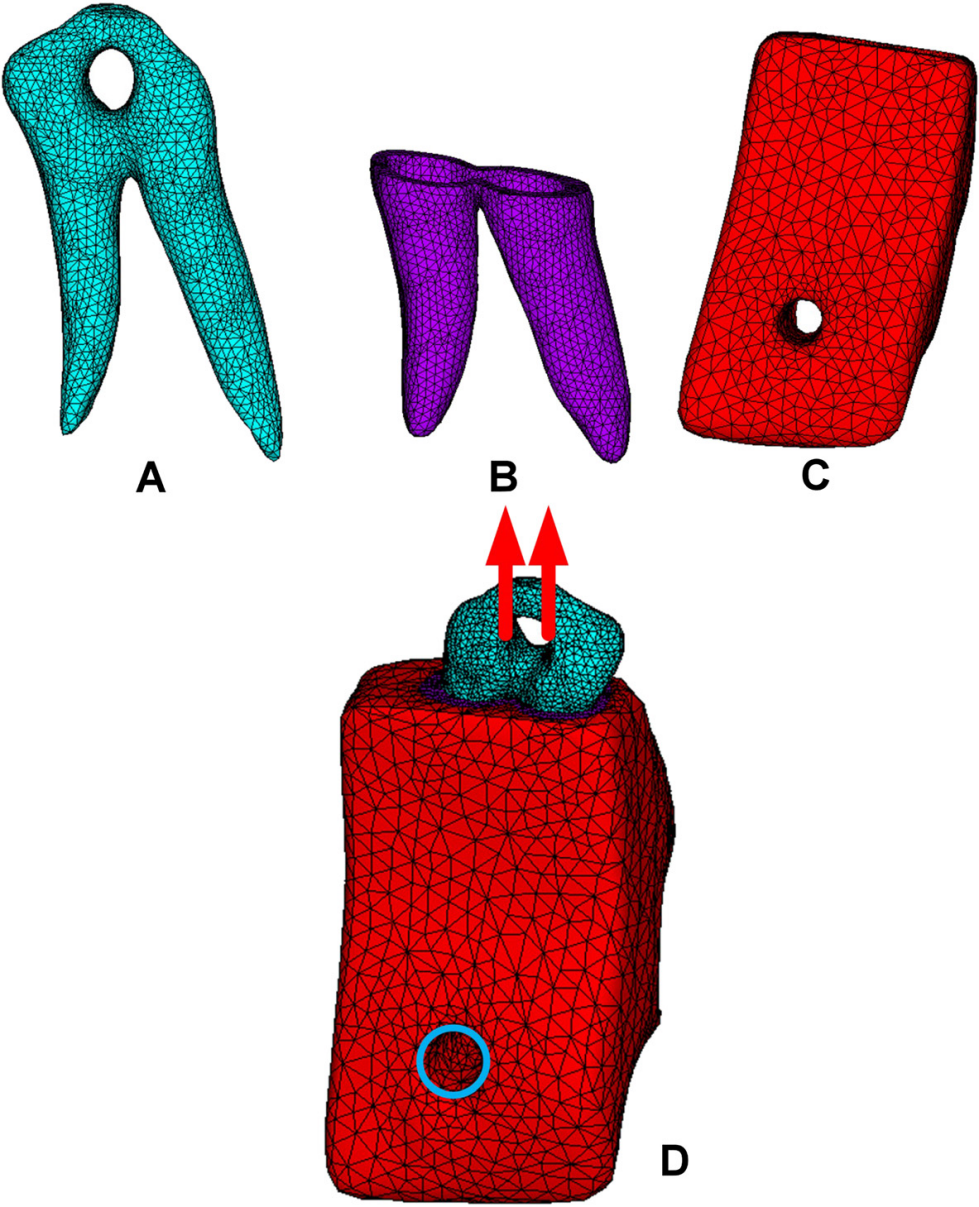
**The finite element models. (A)** tooth, **(B)** periodontal ligament, **(C)** alveolar bone, and **(D)** the entire model with loading and boundary condition.

After building a solid model of the specimen from the STL file, the 3D finite element model (which included the premolar tooth, pulp, PDL, and alveolar bone) was developed in the FE package (ANSYS; ANSYS, Inc., Canonsburg, PA, USA) (Figure 4). A 10-node tetrahedral element (Solid 187) was used in this study. The material properties of the premolar tooth (E = 22000 MPa; *ν* = 0.3) and alveolar bone (E = 1200 MPa; *ν* = 0.3) were defined as isotropic, linear elastic [14]. The material property of the PDL was assumed to be hyperelastic (i.e., a 3 parameters Mooney–Rivlin model). The following formula was used:

$$W=C_{10}\left(\bar{I_{1}}‒3\right)+C_{01}\left(\bar{I_{2}}‒3\right)+C_{11}\left(\bar{I_{1}}‒3\right)\left(\bar{I_{2}}‒3\right)+\frac{1}{d}{\left(J‒1\right)}^{2}$$

in which *C*_*10*_, *C*_*01*_, and *C*_*11*_ are the material constants and *d* is the material incompressibility parameter. $\bar{I_{1}}$ and $\bar{I_{2}}$ are the first and second deviatoric strain invariant. The initial estimation of these material constants (*C*_*10*_ = 0.04 MPa; *C*_*01*_ = 0.02 MPa; *C*_*11*_ *=* 0.04 MPa; and *d* = 0.02), to which previous researchers have referred [11], was established to obtain a proper combination for fitting the experiment results.

The boundary and loading conditions were followed by the experiments. The surface nodes of the perforated hole in the alveolar bone were fixed in every direction and the loadings, according to the experimental results, were applied to the surface nodes of the perforated hole in the tooth. The all interfaces between tissues were continuous to ensure the loading could be properly transmitted.

## Results

Table 1 showed the alveolar bone and tooth volumes at each load step. Volume change variations, which were reconstructed and computed from the serial CT images by STL registration, were all less than 1%. This result implied that the alveolar bone and tooth remained mostly undeformed during loading (i.e., the displacement was primarily caused by the PDL). In addition, a 4 × 4 transformation matrix (representing the translational and rotational information of the tooth) could be acquired from the STL registration. The results of the transformation matrix at each load step approximated the identity matrix which the main diagonal elements are 1’s and all the remaining elements are 0’s. This result implied that a rotational effect could be ignored, whereas the translational term in the occlusal-gingival direction of the transformation matrix could be treated as the real displacement of the tooth. The actual displacement of the tooth could be obtained from the transformation matrix. These results demonstrated that the PDL nearly possesses the bilinear characteristic of stiffness, which increases with the applied force.

**Table 1:** The variations in the volume of the alveolar bone and the tooth at each load step (relative to zero force and displacement)

**Applied Displacement**	**Alveolar Bone Volume (mm**^**3**^**)**	**Error (%)**	**Tooth Volume (mm**^**3**^**)**	**Error (%)**
0 mm	4117.65	–	322.14	–
0.17 mm	4085.32	0.7	323.93	0.6
0.34 mm	4080.62	0.8	324.01	0.6
0.60 mm	4088.57	0.7	323.24	0.3
0.86 mm	4080.59	0.9	323.33	0.4
1.28 mm	4106.22	0.3	322.53	0.1

**Figure 5:**
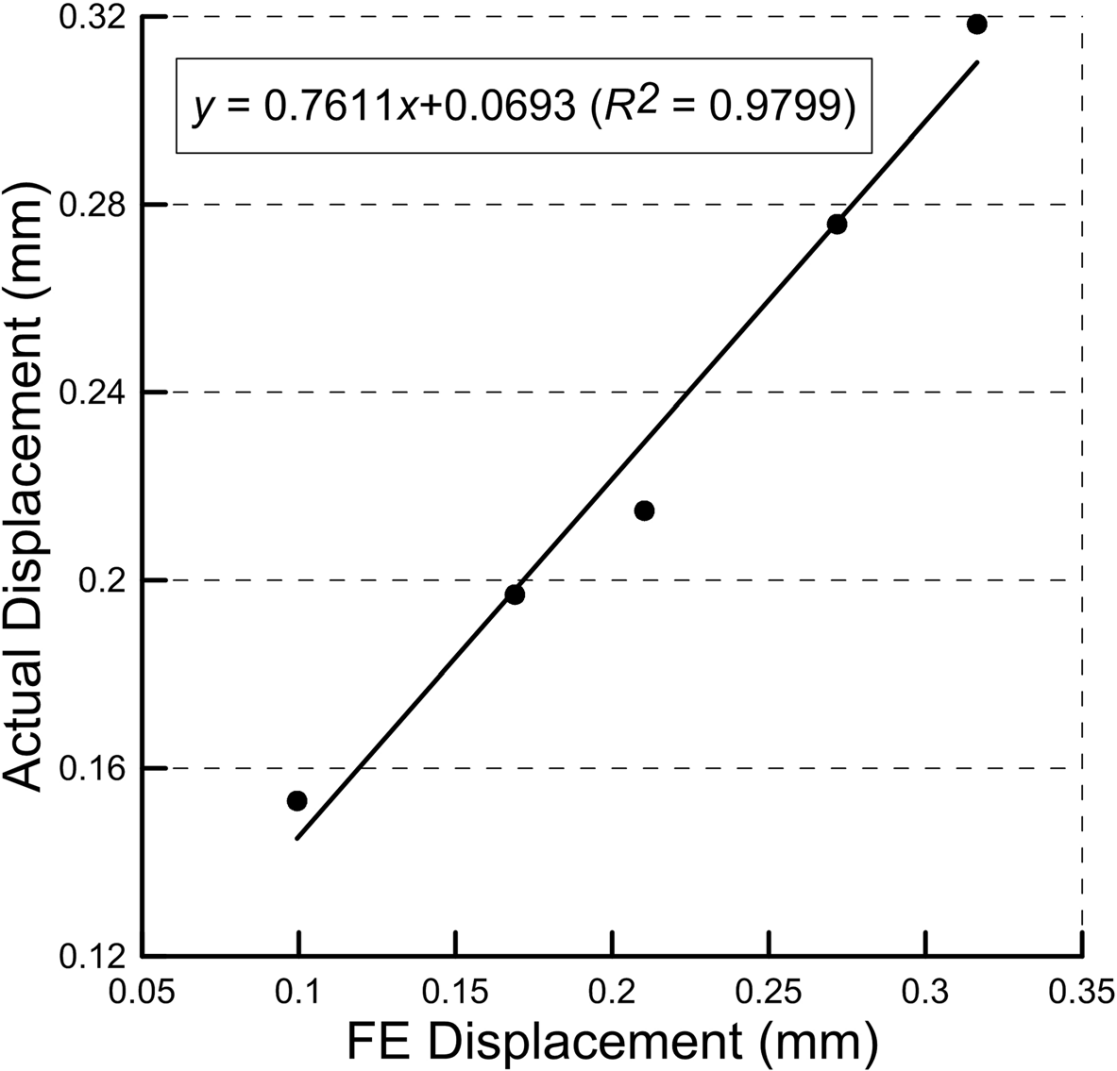
Linear regression of the experimental (i.e., actual) displacement and the finite element (FE) estimated displacement.

The material constants *C*_*10*_, *C*_*01*_, *C*_*11,*_ and *d* were obtained after trial-and-error FE analysis. Through the trial-and-error process, we found that the constants *C*_*11*_ and *d* had a greater effect on the biomechanical properties of the PDL. At the end of the analysis, all material constants were determined as follows: *C*_*10*_ = 1 × 10^−5^ MPa; *C*_*01*_ = 1 × 10^−5^ MPa; *C*_*11*_ *=* 0.1 MPa; and *d* = 4. Figure 5 demonstrates the linear regression of the experimental displacement and the FE estimated displacement. The coefficient of determination was 0.98.

## Discussion

In this study, micro CT was demonstrated to be a useful tool for measuring the biomechanical behavior of the periodontal ligament. This approach can be used to determine the response of the PDL according to the entire displacement with external stimuli applied to the tooth. Many studies have investigated the biomechanical behavior of the PDL by using specimens from animals such as rats, rabbits, and pigs. [4,7,21,22]. Animal specimens are used because intact specimens of the tooth and adjacent alveolar bone from human cadavers are difficult to harvest. This study preserved the intact porcine PDL and adjacent bone for the investigation of PDL biomechanics. Furthermore, using the custom-made apparatus in the micro-CT environment made it possible to simultaneously record the displacement and the corresponding force of the tooth and the PDL. This setup may create an *in vitro* environment that is biomechanically compatible with the loading experienced by the tooth and PDL during occlusion.

**Figure 6:**
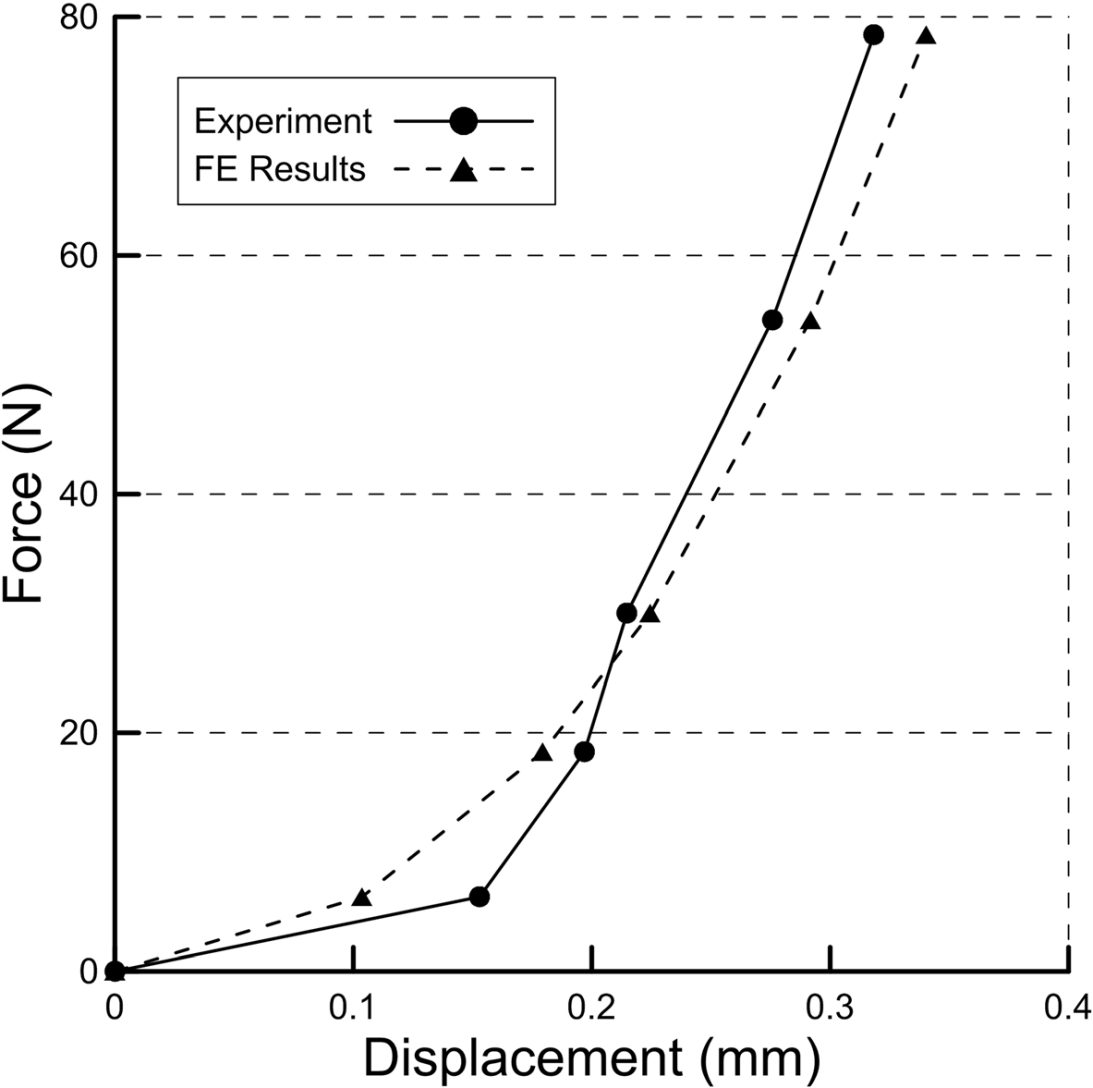
The force-displacement results of the experiment and the finite element (FE) analysis.

This study applied a hyperelastic material model to simulate the mechanical behavior of the PDL. The displacement results between the *in vitro* experiment and the finite element analysis showed a high correlation (Figure 5), which implied the force-displacement results from FE analysis were similar to that from the experiment. This study provided a feasible approach to evaluate the holistic material model of the PDL. The difference between the estimated displacement and the experimental displacement was less than 15%. The force-displacement results also demonstrated that the PDL approximately obeyed hyperelastic behavior (Figure 6). However, the coefficients used in this study were incongruous to those reported in previous studies [11]. This incongruity may be because the experimental and finite element models used in this study were based on the intact PDL of the premolar tooth. Besides, only one tooth was adopted in this study for comparison. Nevertheless, micro CT still provided a feasible modality to measure the biomechanical behavior of the PDL.

Although it is easier to apply a simple linear elastic model, this assumption was not suitable for the soft tissue, which was well-known as a nonlinear material. The general behavior of the soft tissue was divided into three regions: toe, linear and failure regions. When the loading of the soft tissue increased initially, the stiffness of the toe region was low; the linear region was identified after the toe region and had a higher stiffness than the toe region; then the soft tissue failed eventually. It is not easy to clarify the transition zone between each region. Furthermore, the viscoelastic model was also not suitable in this study because the experimental results did not reveal the time-dependent characteristic of the PDL. Therefore, the hyperelastic model was more favorable to fit the nonlinear force-displacement behavior of the PDL.

The results of previous studies represented the local characteristic of the PDL instead of the whole tooth structure; hence, the biomechanical behavior would greatly differ among these studies. Therefore, finding a suitable constitutive model of the PDL is an important issue in dental biomechanics, especially in the development of prosthodontics and orthodontics. However, the parameters reported in this study resulted in greater error when a small force was applied. In addition, each tooth might have its own constitutive equation according to the morphology; therefore, the parameters may still need further validation.

From the sectional CT images, we observed that the transversal thickness of the PDL differed, based on the location of the tooth. The transversal thickness of the PDL was greater around the alveolar crest and the apical region than in other regions. Previous studies have also found similar results [23,24]. In addition, most specimens were sliced transversely into a thin plate for performing a shear test [24], which could only represent the local biomechanical behavior of the PDL. Therefore, this study demonstrated the global biomechanical behavior of the PDL and may have greater differences in comparison to other studies. Furthermore, because multiple roots of a tooth could disturb the biomechanical performance of the PDL during occlusal pressure, this study applied a tension force to highlight the independent effect of the PDL. The resistance force increased dramatically with the displacement after passing the toe region, based on the experimental results (Figure 6). This would explain the protective mechanism that prevents the tooth from excessive extrusion.

Testing in the micro-CT environment has several advantages compared to the previous experiments. First, it is a nondestructive modality for performing biomechanical testing. Further biological or physiological analysis can be performed because the PDL specimen remains intact. Second, micro or local observations can be accomplished because micro-CT has a higher resolution than traditional modalities such as CT, magnetic resonance imaging, or ultrasound images. Analysis of the intact PDL or observation of internal morphology is available by 3D model reconstruction because no permanent damage occurs within the physiological loading range.

Bone remodeling is an important process in orthodontic biomechanics. Studies report a direct correlation of stress–strain fields in the PDL with alveolar bone resorption [25,26]. While PDL can be a direct effector, the response of bone to mechanical load could be a delayed effect that is necessary for tooth translation. Instead of a time-dependent (i.e., viscoelastic) characteristic of the PDL, our results showed good agreement between the numerical simulation and the experiment. This implied that the hyperelastic constitutive model is practical for investigating the biomechanical response of the PDL. The mechanobiological interaction of the PDL and alveolar bone nevertheless remains indeterminable.

Our results demonstrated that the PDL is suitable for simulating hyperelastic material; however, some limitations still need to be depicted. Because of the scattering effect of the micro-CT, the material selected for the custom-made apparatus should be taken in account to eliminate its influence in image registration. The experimental results and the hyperelastic model can well interpret the steady state of the PDL after loading because the entire scanning process requires more than 30 minutes. More samples under various loading modes are required for a complete evaluation of the biomechanical property of the PDL. The detailed tooth structures were not modelled in this study, such as enamel, dentine and pulp. The Young’s modulus and stiffness of the anatomical layer of enamel and dentine was much greater than those of the PDL. In addition, the volume of pulp took a very small part of the entire tooth. Furthermore, from the results of micro-CT reconstruction, the tooth and bone was approximately undeformed. Therefore, the lack of these layers in FE model would not affect the results much. This study excluded the behavior of the PDL at a high strain rate. For this study, only one specimen of a porcine mandibular premolar tooth was harvested. However, finding a suitable constant set of the hyperelastic model was a time-consuming task. Besides, the individual difference was existed in every sample harvested from the porcine. Although more specimens may have enhanced the reliability of the experimental and numerical results, this study successfully established a feasible approach to predict the force-displacement relation of the PDL in a holistic observation.

## Conclusion

Micro-computed tomography, which performs nondestructive measurements, is capable of obtaining the biomechanical behavior of the PDL. Using the hyperelastic characteristic as the constitutive model may properly predict the force-displacement relation of the PDL after loading.

## Competing interests

The authors declare that they have no competing interests.

## Authors’ contributions

CHC, YNL and TSL conceived and designed the experiments; YNL and YHS collect the sample for experiments. YHS and YHH performed the experiments and analyzed the data; YHS, YHH and TSL wrote the manuscript; and all of the authors read and approved the final version of manuscript.

## References

[B1] Proffit WR Fields HW Sarver DM Contemporary Orthodontics 2013 5 St. Louis, MO: Elsevier/Mosby

[B2] Toms SR Lemons JE Bartolucci AA Eberhardt AW Nonlinear stress–strain behavior of periodontal ligament under orthodontic loading Am J Orthod Dentofacial Orthop 2002 122 174 179 12165771 10.1067/mod.2002.124997

[B3] Dorow C Krstin N Sander F-G Determination of the mechanical properties of the periodontal ligament in a uniaxial tensional experiment J Orofac Orthop 2003 64 100 107 12649706 10.1007/s00056-003-0225-7

[B4] Natali AN Carniel EL Pavan PG Bourauel C Ziegler A Keilig L Experimental-numerical analysis of minipig’s multi-rooted teeth J Biomech 2007 40 1701 1708 17074355 10.1016/j.jbiomech.2006.08.011

[B5] Tohill R Hien M McGuinness N Chung L Reuben RL Sloten J, Verdonck P, Nyssen M, Haueisen J Measurement Of The Short-Term Viscoelastic Properties Of The Periodontal Ligament Using Stress Relaxation4th European Conference of the International Federation for Medical and Biological Engineering; 23–27 November, 2008 2009 Belgium: Springer 1467 1470

[B6] Rees JS Jacobsen PH Elastic modulus of the periodontal ligament Biomaterials 1997 18 995 999 9212195 10.1016/s0142-9612(97)00021-5

[B7] Kawarizadeh A Bourauel C Jager A Experimental and numerical determination of initial tooth mobility and material properties of the periodontal ligament in rat molar specimens Eur J Orthod 2003 25 569 578 14700262 10.1093/ejo/25.6.569

[B8] Poppe M Bourauel C Jager A Determination of the elasticity parameters of the human periodontal ligament and the location of the center of resistance of single-rooted teeth a study of autopsy specimens and their conversion into finite element models J Orofac Orthop 2002 63 358 370 12297965 10.1007/s00056-002-0067-8

[B9] Ziegler A Keilig L Kawarizadeh A Jager A Bourauel C Numerical simulation of the biomechanical behaviour of multi-rooted teeth Eur J Orthod 2005 27 333 339 15961572 10.1093/ejo/cji020

[B10] Komatsu K Shibata T Shimada A Viidik A Chiba M Age-related and regional differences in the stress–strain and stress–relaxation behaviours of the rat incisor periodontal ligament J Biomech 2004 37 1097 1106 15165880 10.1016/j.jbiomech.2003.11.013

[B11] Natali AN Pavan PG Carniel EL Dorow C Viscoelastic response of the periodontal ligament: an experimental–numerical analysis Connect Tissue Res 2004 45 222 230 15763931 10.1080/03008200490885742

[B12] van Driel WD van Leeuwen EJ Von den Hoff JW Maltha JC Kuijpers-Jagtman AM Time-dependent mechanical behaviour of the periodontal ligament Proc Inst Mech Eng [H] 2000 214 497 504 10.1243/0954411001535525 11109857

[B13] Qian L Todo M Morita Y Matsushita Y Koyano K Deformation analysis of the periodontium considering the viscoelasticity of the periodontal ligament Dent Mater 2009 25 1285 1292 19560807 10.1016/j.dental.2009.03.014

[B14] Natali AN Pavan PG Scarpa C Numerical analysis of tooth mobility: formulation of a non-linear constitutive law for the periodontal ligament Dent Mater 2004 20 623 629 15236936 10.1016/j.dental.2003.08.003

[B15] Wood SA Strait DS Dumont ER Ross CF Grosse IR The effects of modeling simplifications on craniofacial finite element models: the alveoli (tooth sockets) and periodontal ligaments J Biomech 2011 44 1831 1838 21592483 10.1016/j.jbiomech.2011.03.022

[B16] Verma P Love RM A micro CT study of the mesiobuccal root canal morphology of the maxillary first molar tooth Int Endod J 2011 44 210 217 20880136 10.1111/j.1365-2591.2010.01800.x

[B17] Naveh GRS Shahar R Brumfeld V Weiner S Tooth movements are guided by specific contact areas between the tooth root and the jaw bone: a dynamic 3D microCT study of the rat molar J Struct Biol 2012 177 477 483 22138090 10.1016/j.jsb.2011.11.019

[B18] Chen G Fan W Mishra S El-Atem A Schuetz MA Xiao Y Tooth fracture risk analysis based on a new finite element dental structure models using micro-CT data Comput Biol Med 2012 42 957 963 22901677 10.1016/j.compbiomed.2012.07.006

[B19] Lin JD Özcoban H Greene JP Jang AT Djomehri SI Fahey KP Hunter LL Schneider GA Ho SP Biomechanics of a bone–periodontal ligament–tooth fibrous joint J Biomech 2013 46 443 449 23219279 10.1016/j.jbiomech.2012.11.010 PMC3690590

[B20] Cho E Sadr A Inai N Tagami J Evaluation of resin composite polymerization by three dimensional micro-CT imaging and nanoindentation Dent Mater 2011 27 1070 1078 21820729 10.1016/j.dental.2011.07.008

[B21] Dorow C Krstin N Sander F-G Experiments to determine the material properties of the periodontal ligament J Orofac Orthop 2002 63 94 104 12506782 10.1007/s00056-002-0107-4

[B22] Komatsu K Chiba M Synchronous recording of load-deformation behaviour and polarized light-microscopic images of the rabbit incisor periodontal ligament during tensile loading Arch Oral Biol 2001 46 929 937 11451407 10.1016/s0003-9969(01)00054-1

[B23] Hurng JM Kurylo MP Marshall GW Webb SM Ryder MI Ho SP Discontinuities in the human bone-PDL-cementum complex Biomaterials 2011 32 7106 7117 21774982 10.1016/j.biomaterials.2011.06.021 PMC3185383

[B24] Toms SR Dakin GJ Lemons JE Eberhardt AW Quasi-linear viscoelastic behavior of the human periodontal ligament J Biomech 2002 35 1411 1415 12231287 10.1016/s0021-9290(02)00166-5

[B25] Katona TR Paydar NH Akay HU Roberts WE Stress analysis of bone modelling response to rat molar orthodontics J Biomech 1995 28 27 38 7852439 10.1016/0021-9290(95)80004-2

[B26] Kawarizadeh A Bourauel C Zhang D Götz W Jäger A Correlation of stress and strain profiles and the distribution of osteoclastic cells induced by orthodontic loading in rat Eur J Oral Sci 2004 112 140 147 15056111 10.1111/j.1600-0722.2004.00116.x

